# Risk Factors for, and Prediction of, Shoulder Pain in Young Badminton Players: A Prospective Cohort Study

**DOI:** 10.3390/ijerph192013095

**Published:** 2022-10-12

**Authors:** Antonio Cejudo

**Affiliations:** 1 Department of Physical Activity and Sport, Faculty of Sport Sciences, CEIR Campus Mare Nostrum (CMN), University of Murcia, 30720 Murcia, Spain; antonio.cejudo@um.es; Tel.: +34-868-888-430; 2 Locomotor System and Sport Research Group (E0B5-07), University of Murcia, 30720 Murcia, Spain

**Keywords:** racquet sport, shoulder injury, overuse injury, muscle tightness, glenohumeral internal rotation deficit

## Abstract

Background: Shoulder pain (SP) caused by hitting the shuttlecock is common in young badminton players. The objectives of the present study were to predict the risk factors for SP in young badminton players, and to determine the optimal risk factor cut-off that best discriminates those players who are at higher risk of suffering from SP. Methods: A prospective cohort study was conducted with 45 under-17 badminton players who participated in the Spanish Championship. Data were collected on anthropometric age, sports history, sagittal spinal curves, range of motion (ROM) and maximum isometric strength of shoulder. After 12 months, players completed a SP history questionnaire. Bayesian Student’s *t*-analysis, binary logistic regression analysis and ROC analysis were performed. Results: Overall, 18 (47.4%) players reported at least one episode of SP. The shoulder internal rotation (SIR) ROM showed the strongest association (OR = 1.122; *p* = 0.035) with SP. The SIR ROM has an excellent ability to discriminate players at increased risk for SP (*p* = 0.001). The optimal cut-off for SIR ROM, which predicts players with an 81% probability of developing SP, was set at 55° (sensitivity = 75.0%, specificity = 83.3%). Conclusions: The young badminton players who had a shoulder internal rotation ROM of 55° or less have a higher risk of SP one year later.

## 1. Introduction

Badminton is one of the most popular sports, characterised by a temporal structure in which short, high-intensity intervals of exertion alternate with short rests in a ratio of about 1:3 [1]. During the exchange of strokes, badminton players perform complex and varied technical–tactical movements, such as multidirectional strides and movements, rapid changes of direction, jumps and explosive strokes with the shuttlecock from different positions [2]. Although badminton is considered a relatively safe sport, shoulder injuries are very common among both recreational and competitive players [3,4].

Epidemiological studies have shown that the most common injuries among badminton players are overuse injuries [5,6,7,8,9,10], with the proportion of overuse injuries being three times higher than that of trauma injuries [6,7]. Most scientific studies have found a prevalence of overuse injuries of more than 71% in different age groups and competitive levels, e.g., recreational players [5], competitive players at national level [5,7] and competitive players at international level [8]. Of the total overuse injuries, more than 11% (between 11% and 37%) are localised in the shoulder [2,5,6,9,11]. The prevalence of SP is higher in rowing, handball and wrestling athletes and lower in volleyball, basketball and swimming athletes [12]. The incidence of shoulder injuries is reported to be 0.3 to 0.5 (0.33 elite senior; 0.50 elite junior) injuries per 1000 h of training and competition [9]. The most common overuse injuries diagnosed in badminton players are subacromial syndrome or rotator cuff impingement [4,11], rotator cuff tendinopathy [5,8,13], and biceps brachii [13]. The scapulothoracic instability or dyskinesia [3,4], shoulder dislocation [14] and acromioclavicular subluxation [9] have also been diagnosed. It should be emphasised that subacromial syndrome, anterior instability and scapulothoracic dyskinesia are considered the most common causes of shoulder pain (SP) in badminton players [3,4,11,15].

In the scientific literature, studies on injuries and trauma related to badminton predominate [5,8,9,13,16]; however, some authors report that there are few scientific studies on the relationship between non-serious injuries such as overuse injuries and the history of SP [17]. Shoulder pain is a very common musculoskeletal complaint in recreational [11], national [17], and international [4] competitive players. At an international championship, 188 badminton players voluntarily completed a SP survey, and the survey results showed a prevalence of a history of SP of 52% [4]. Similar results (52%) were found in a survey of past and current SP related to badminton among 99 amateur players (mean age 43 years) participating in a Swedish national championship [11]. In young badminton players, a prevalence of SP of 27.6% was found in 151 junior players (14 to 18 years) who participated in the European Junior Championships [18]. At a Japanese national championship, 53.3% of 1002 badminton players aged 12–22 years reported a badminton-associated SP [17]. Of particular concern in the findings cited above is the early onset of SP in young (12–18 years) badminton players [17]. Furthermore, more than one third of young players show permanent SP during badminton training [3], although this will lead to chronic SP and overuse injuries in the future [19]. Therefore, it is believed that the use of SP preventive measures in young badminton players can be an important goal to avoid overuse injuries and SP [17].

The physical–technical demands of badminton predispose players to suffer SP. The sport requires a significant number (30%) of overhead shuttlecock strokes such as clear, smash and drop shots, which are executed with short and explosive movements [1,2,3]. High strokes request a large range of motion (ROM) of shoulder external rotation and abduction [2,4,17] and internal rotation and anterior tilt of the scapula [20]. In addition, the shoulder acts as a transmitter of forces generated by the lower limbs and trunk to the arm during the steering wheel impact movement. These forces, as well as the repeated overhead strokes, therefore lead to significant stresses on the various joint tissues of the shoulder [3]. These high physical–technical demands for strength, power, flexibility and dynamic structural stability lead to certain negative joint adaptations in the shoulder [3,17]. Badminton players with SP have been found to have reduced ROM shoulder abduction on the dominant side [4] and homolateral and bilateral force imbalances in the acceleration or striking and deceleration phases [3,21,22]. Given the high prevalence of SP and the above-mentioned physical-technical demands of badminton, it seems necessary to ensure the participation of young players without SP in national and international competitions. To this end, identifying risk factors for SP could be an excellent way to develop and implement early intervention with preventive measures and rehabilitation based on scientific evidence. Currently, modifiable risk factors that predispose to SP are still unknown [3].

The main objective of the present study was to predict the risk factors for SP in young badminton players participating in the Spanish Championships. A second objective was to determine the optimal risk factor cut-off value that best discriminates those players who are at higher risk of suffering from SP.

The hypothesis is that dorsal hyperkyphosis, limited shoulder ROM and shoulder weakness predict SP in young badminton players. The optimal cut-off value will be lower than the normative values proposed for the non-athletic population.

## 2. Materials and Methods

### 2.1. Ethics Committee Approval Statement

This observational study was developed according to the guidelines and checklist of the STROBE (Strengthening the Reporting of Observational studies in Epidemiology) Initiative Statement [23,24]. The study was approved by the Institutional Review Board of the University of Jaén (Spain) before experiment was started (Reg. Code JUN 18/10 TES) and that has been conducted in accordance with the principles set forth in the Helsinki Declaration.

### 2.2. Study Design

**Figure 1:**
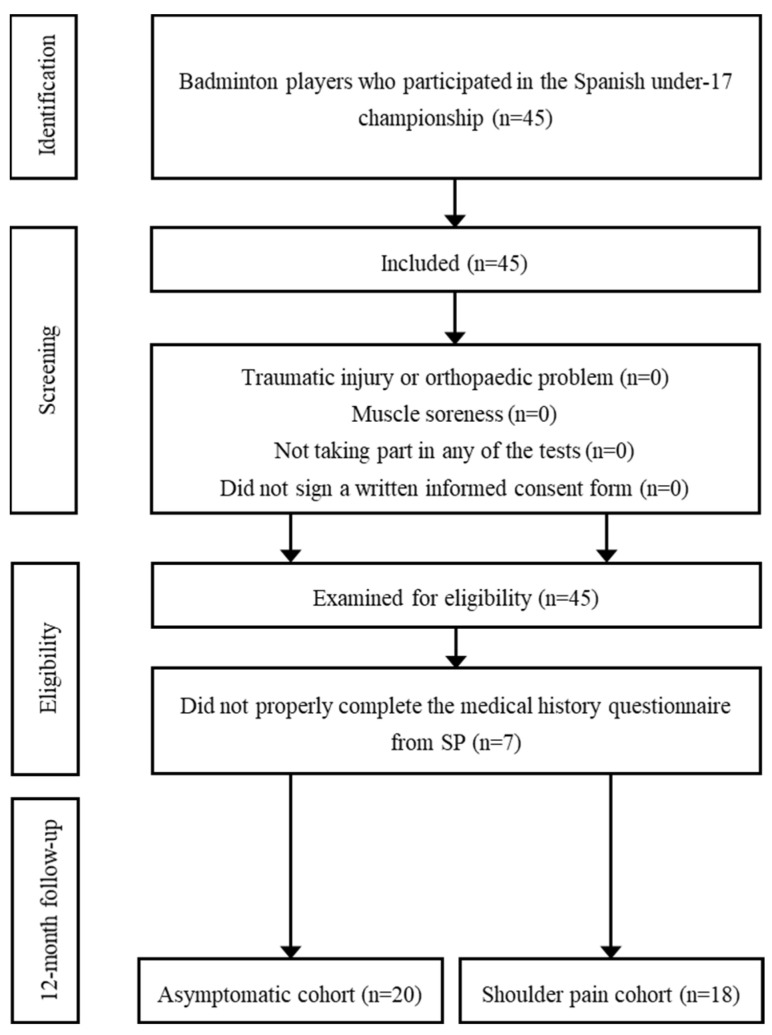
STROBE Flow Diagram.

A case–control study embedded in a prospective cohort study was conducted to predict risk factors for SP in 45 young badminton players and to establish cut-off values for risk factors that distinguish players at increased risk for SP (Figure 1).

Before participating in the study, the directors of the Spanish Championship, the technical teams of the autonomous teams and the parents/guardians of the players were informed verbally and in writing about the experimental procedure and the possible risks, and subsequently gave their written informed consent. The badminton players were recruited from the Spanish Championship (under-17). One day before the start of the Spanish Championship, after the control session of the players by the championship organisation, a test familiarisation session was conducted with the players. At the Spanish Championship, the independent variables were measured (shoulder ROM, shoulder maximum isometric strength (MIS) and the sagittal spinal curves (SSC)); information was also collected on confounding variables such as anthropometric measures, badminton experience and the players’ level of competition. Measurement of these variables was performed before daily badminton competition in a sports hall under standard conditions of 24–25 °C. Prior to testing, players performed a general dynamic warm-up following the recommendations of previous studies [25], and then a specific warm-up was performed prior to shoulder strength assessment [26,27,28]. The measurements were performed by two senior examiners with more than 15 years of experience in musculoskeletal measurement (Ph.D. in Sports Science). The tests were performed in random order using the software http://www.randomizer.org to avoid bias in the results due to a specific order. Each test was performed three times with each player and the mean of the two closest measurements was used for subsequent statistical analysis. The dominant shoulder was defined as the side of the body of the hand with which the player grips the badminton racket. Finally, a prospective measurement of SP was conducted after 12 months (outcome) by completing a questionnaire via telephone survey. The players were thus exposed to 12 months of training and competitions, including the Spanish championship (Exposure).

### 2.3. Participants

The sample consisted of 45 under-17 badminton players (aged 16 to 17) selected by the coach of the regional federation to participate in the Spanish Championship “Campeonato de España de Selecciones Autonómicas en Edad Escolar e Inclusivo” (San Lorenzo de El Escorial, Spain). All players were selected as top 3 players by the coach of the regional federation. The players had at least three years of badminton experience and trained at least three days per week and one hour per badminton training session.

Players who had suffered a traumatic injury or an orthopaedic problem of the upper limbs or trunk in the last six months that could affect the anthropometric characteristics or the results of the evaluation tests were excluded, as were players who had muscle soreness after competition. Players who did not participate in any of the tests, did not properly complete the medical history questionnaire of SP or did not sign a written informed consent form prior to the examination were not included in the statistical analysis. The specific criteria for cases were those players who developed SP in the 12 months following the study evaluation. A double-blind method was developed in which players and examiners did not know which participants were assigned to each cohort (SP group—cases—or asymptomatic group—control).

### 2.4. Examiners

The tests were administered by two experienced examiners who developed specific competencies for each test procedure. In general, the main examiner informed the player about the steps of the procedure. Then, the main examiner performed the movement of the test and the measurement with the measuring instrument, while the assistant examiner avoided compensatory movements (ROM and MIS) or assisted the main examiner with some competencies (SSC). An assistant familiar with the procedure recorded the data on the record sheet. In a study of 12 active young adults (two assessment sessions 24 h apart) using a single-blinded method, the assessors demonstrated excellent reliability, with an intraclass correlation coefficient at 95% probability (ICC_95%_) of greater than 0.89 and a minimum detectable change at 95% probability (MCD_95%_) of no more than 5.3° (SSC and ROM) and 10.2 newtons (MIS).

### 2.5. Procedure Study

All players received a comprehensive theoretical-practical explanation of the assessment tests in the familiarization session. Previous research studies showed reasonable test–retest reliability values for ROM (CCI_95%_ = 0.88 to 0.96; MCD_95%_ = 3.7° to 7.2°) [29], MIS (CCI_95%_ = 0.89 to 0.96; MCD_95%_ = 8.6 to 10.8N) [26], and SSC (CCI_95%_ = 0.93 to 0.98; MCD_95%_ = 0.85° to 2.3°) [30,31], which were used in the present study.

#### 2.5.1. Questionnaire

The players completed a questionnaire consisting of three parts: (1) age and anthropometric data, (2) sports history and (3) SP history. After the control session of the players in the Spanish Championship, the players filled in 2/3 of the questionnaire (age, anthropometric data and sport history of the players).

Anthropometric data were measured using standardised techniques according to the ISAK protocol [32]. Height and body mass were measured with a mobile stadiometer (Seca 799; Seca Ltd., Hamburg, Germany) with an accuracy of 0.1 cm and 0.5 kg, respectively. A correction of 0.5 kg was made for clothing weight. Body mass index was calculated from body mass and height by dividing body mass (kg) by height (m) squared.

In the second part of this procedure, the players were asked questions about their sports background, such as years of experience, maximum competition level, current competition category, dominant upper-limb, total weekly training, weekly training frequency, duration of training sessions and average competition duration.

Twelve months after the end of the Spanish Championship, the third part of the questionnaire was completed by telephone survey to collect information on the history of SP. The objective of this part of the questionnaire was to identify players with SP to form the effect or outcome cohort (SP group). The players were asked the following three questions, following the recommendations of previous studies [33,34]:

− Have you ever had shoulder pain in the last 12 months? ☐ No ☐ Yes
− In which shoulder did you have pain? ☐ Dominant ☐ Non-dominant ☐ Both
− How severe was the shoulder pain? ☐ Minimal injury (2 to 3 days without training and competition), ☐ Minor injury (4 to 7 days without training or competition), ☐ Moderate injury (8 to 28 days without training or competition) or ☐ Severe injury (more than 28 days without training or competition).

Shoulder pain was considered if the player had an injury that caused an absence from training and competition of more than three days (minor injury).

#### 2.5.2. Sagittal Spinal Curves Assessment

**Figure 2:**
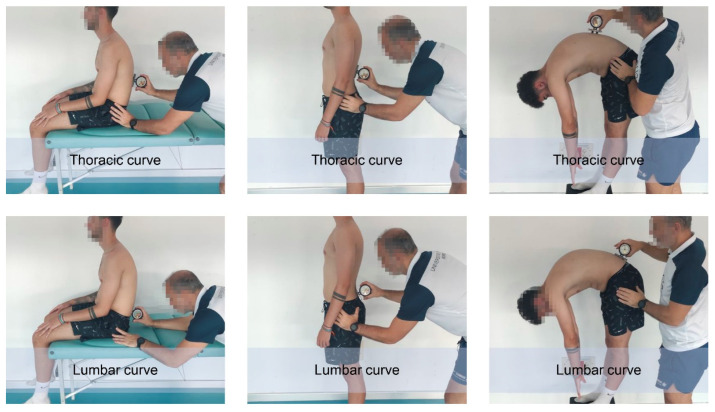
Assessment of spinal sagittal curves.

Thoracic and lumbar sagittal spinal curves were measured in the slumped sitting (SSP), the relaxed standing (RSP) and the maximum trunk forward flexion (TFP) postures according to the method described by Santonja et al. [35]. An inclinometer (ISOMED Unilevel, Inc., Portland, OR, USA) was used to measure the angle of both curves (Figure 2).

Before measuring the sagittal spinal curves, the first thoracic vertebra (T1), the twelfth thoracic vertebra (T12) and the fifth lumbar vertebra (L5) were marked on the skin to determine the curves in RSP and MTFP. Measurement of sagittal spinal curves in both postures requires these markings to distinguish or delineate thoracic curve from lumbar curve [35,36,37].

To measure the curves in the RSP, the player adopted a relaxed standing posture. The inclinometer was placed at T1 and calibrated to 0°. Then, the inclinometer travelled down the spine until it reached the maximum value of the thoracic curve. Finally, the data was read and recorded. At this step, the inclinometer was calibrated again to 0°. Then, the inclinometer moved down the spine until it reached the maximum value of the lumbar curve. Finally, the data were read and recorded [38].

To measure the curves in SSP, the player sat on the table in a slump posture with both thighs fully supported, forearms resting on the thighs, knees bent and feet off the floor. The inclinometer was placed at T1 and calibrated to 0°; then the inclinometer was placed at T12. At this time, the thoracic curve data was read and recorded. The inclinometer was then recalibrated to 0° at T12. The inclinometer was then placed at L5 to read and record the lumbar curve value.

In TFP, curves were measured similarly to SSP, except that the player adopted a TFP while the lower limbs were in a neutral position, i.e., with knees extended [35].

#### 2.5.3. Shoulder Range of Motion Assessment

**Figure 3:**
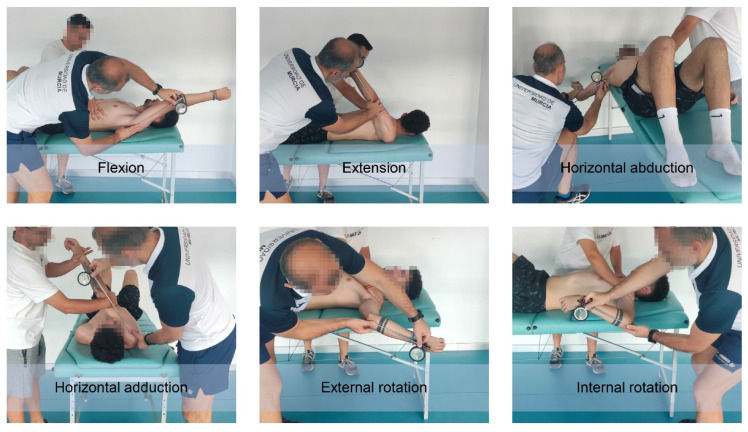
Assessment of shoulder range of motion.

The main shoulder movements (flexion, extension, internal rotation, external rotation, horizontal abduction and horizontal adduction) ROM of the dominant and non-dominant limbs (Figure 3) were quantified in their maximum passive expression using the ROM-SPORT II battery [26,29].

An ISOMED Unilevel inclinometer (ISOMED Unilevel, Inc., Portland, OR, USA) was used to measure the angle of the ROM based on inclinometer techniques [37,39]. The measurement instrument was calibrated at 0° with vertical (internal and external rotation) or horizontal (flexion, extension, horizontal adduction and horizontal abduction) gravity line before evaluation. The telescopic arm of the inclinometer is placed over the arm, forearm or both (depending on the type of movement or test) and follows its bisector line. Then, the main examiner records the angle that the longitudinal axis of the body segment makes with the horizontal or vertical plane [39].

#### 2.5.4. Maximum Isometric Strength of the Shoulder Assessment

**Figure 4:**
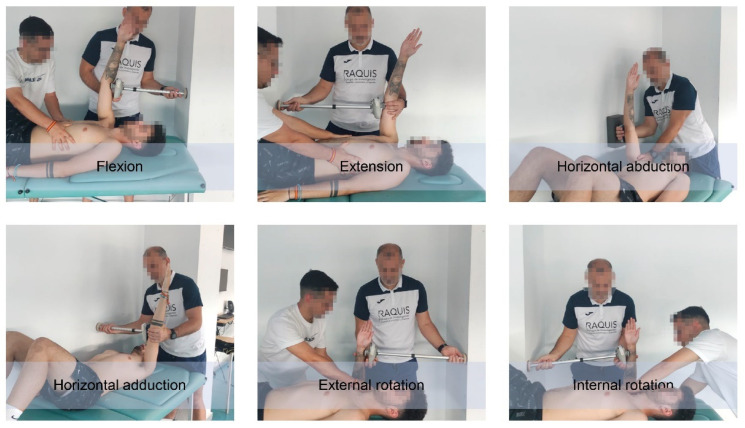
Assessment of maximum isometric strength of shoulder.

Prior to the maximum isometric strength (MIS) measurements, players performed two progressive repetitions up to 80% of MIF as a specific warm-up [28]. The MIS of the main shoulder movements (flexion, extension, horizontal abduction, horizontal adduction, internal rotation and external rotation) of the dominant and non-dominant limbs was measured (Figure 4) following a previously described procedure [26].

A Lafayette hand-held dynamometer (Lafayette Instrument Company, Lafayette, IN, USA) and an extendable arm were used to measure MIS to develop isometric strength [26].

Lying supine on a table, the player applied the MIS on a wall. For this purpose, the main examiner placed the dynamometer directly or the extended arm when he could determine the distance between the player’s upper limb and the wall. The dynamometer was placed in the distal region of the arm during all movements except shoulder rotation to avoid flexion or extension compensations of the arm. Players were given 5 s to apply the MIS [26]. The main examiner instructed the players to gradually apply strength until the MIS was reached before the 5 s avoiding compensatory movements that lead to additional MIS of other muscles. The dynamometer emitted a tone at the beginning and end of the period. The peak MIS exerted (N) and the time of maximum MIS expression (s) were automatically recorded by the mentioned measuring instrument.

### 2.6. Statistical Analysis

Previously, the sample size required for this study was determined a priori by establishing a reasonable power (1-ß probability of error). The effect size was obtained from the significant difference of the shoulder ROM between the SP group and the asymptomatic group in a sample of athletes [29]. The software package G*Power version 3.1.9.4 (Heinrich Heine University of Düsseldorf, Düsseldorf, Germany) was used for the calculation.

Statistical analyses were performed using the software JASP version 0.14.01 (JASP team of the University of Amsterdam, Amsterdam, The Netherlands). Due to the final sample size, it was decided to use Bayesian statistics instead of frequentist statistics. Bayesian inference has recently been proposed as a more robust alternative to traditional frequentist statistics (based on confidence intervals and *p*-values) for hypothesis testing. This method is based on quantifying the relative degree of evidence for two competing hypotheses, the null hypothesis (H0) versus the alternative hypothesis (H1), using the Bayes factor (BF_01_–BF_10_) [40,41]. Hypothesis H1 and H0 refer to the probability that the comparison of the evaluated variables is different and equal, respectively.

Normality of the data was confirmed using the Shapiro–Wilk test. Abnormally distributed data showed a Gaussian distribution after log transformation. All continuous data are reported as mean ± standard error and 95% confidence interval.

Differences in ROM and MIS between the shoulder of the dominant side of the body and the non-dominant side of the body were determined using the Bayesian Student’s *t*-test.

The Bayesian Student’s *t*-test for independent samples was applied to determine the comparison of means between the SP group and the asymptomatic group. The BF_10_ was interpreted using the previously proposed evidence categories [42]: <1/100 = extreme evidence for H0, 1/100 to 1/30 = very strong evidence for H0, 1/30 to 1/10 = strong evidence for H0, 1/10 to 1/3 = moderate evidence for H0, 1/3 to 1 anecdotal evidence for H0; 1 to 3 = anecdotal evidence for H1, 3 to 10 = moderate evidence for H1, 10 to 30 = strong evidence for H1, 30 to 100 = very strong evidence for H1, >100 extreme evidence for H1. Models that showed at least moderate evidence with a percentage error > 3 were considered sufficiently robust to describe the main effects. The mean and 95% interval credible of the posterior distribution of the standardised effect size (δ) was calculated (i.e., the population version of Cohen’s d) for comparisons between groups.

Identification of risk factors (age, anthropometry, sports history, sagittal spinal curve, shoulder ROM and shoulder MIS) associated with SP was determined by binary logistic regression analysis using the Enter method. This statistical analysis calculated the sign of the estimate, the standard error, the odds ratio (OR) or odds ratios, z, the *p*-value and the associated 95% confidence intervals (CI).

To determine the optimal cut-off value of the predictor variables, a receiver operating characteristic analysis (ROC) was conducted using the open-source statistical software Jamovi version 1.6.23. The predictive ability of the identified predictors was calculated using the area under the curve (AUC). The AUC value was classified as outstanding (0.90 ≥ AUC < 1.00), excellent (0.80 ≥ AUC < 0.90), acceptable (0.70 ≥ AUC < 0.80), poor (0.50 ≥ AUC < 0.80) and no discrimination (AUC < 0.50) [43]. Second, the optimal cut-off value (or with the highest discriminatory ability) that maximised the ratio between sensitivity and specificity was determined using the Youden index, i.e., the optimal cut-off value that provided the best discriminatory ability between players with SP and asymptomatic players. In addition, the positive predictive value (PSV) and the negative predictive value (NPV) were calculated.

The correlation between the identified predictors (low risk versus high risk for the optimal cut-off value) and SP was determined using Pearson’s chi-square statistic. As well as the magnitude of the association or the effect size according to Cramer’s V.

## 3. Results

A sample size of at least 30 players (effect size = 0.96) was required to achieve a minimum sample power of 80. Seven players did not respond to the telephone survey questionnaire corresponding to the SP history. Thus, a total of 38 players met the inclusion and exclusion criteria. The characteristics of these players were 16.26 ± 0.45 years old, 66.45 ± 9.08 kg body mass, 1.76 ± 0.11 cm body height, 21.74 ± 3.94 kg/m^2^ body mass index, 6.37 ± 1.82 years of badminton experience, 9.76 ± 5.59 h of weekly training, 3.95 ± 1.06 days of weekly training and 2.25 ± 0.70 h of training sessions.

Differences between the two sides of the body were found in the shoulder ROM and MIS. Table 1 shows that the values of the shoulder ROM (flexion, extension, adduction, internal rotation and external rotation) were lower in the dominant shoulder and the values of the shoulder MIS (flexion, extension and internal rotation) were higher than in the non-dominant shoulder.

A total of 18 (47.4%) players reported at least one episode of SP. Table 2 show the comparison between the SP and the asymptomatic groups regarding the variables evaluated (risk factor) in this study. Differences between the 2 groups were only observed in dominant internal rotation ROM (63.20 ± 17.00° versus 51.33 ± 7.03°; BF_10_ = 5.334 [moderate]).

The predictive model built from the variables with differences between groups in Table 3 (ROM: adduction, dominant internal rotation and horizontal abduction; MIS: extension and MIS imbalance of internal rotation) showed an adequate Nagelkerke’s R2 adjustment of 0.491. Of all the variables included in the predictive model, shoulder internal rotation ROM showed the strongest association with SP (OR = 1.122; 95% CI = −0.224 to −0.008; *p* = 0.035). Odds ratios showed that low values of shoulder internal rotation ROM increased the probability of experiencing SP by 12.2%.

**Figure 5:**
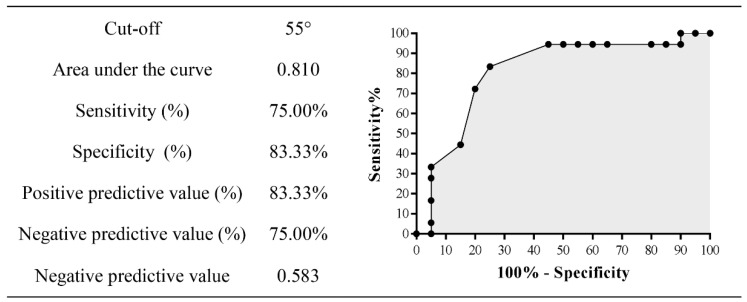
Optimal cut-off value for shoulder internal rotation range of motion.

Shoulder internal rotation ROM showed excellent ability to discriminate (*p* = 0.001; area under the curve [AUC] = 0.810) those players with increased risk for SP. The optimal cut-off value for shoulder internal rotation ROM, which predicts players at increased risk for SP, was set at 55° (sensitivity = 75.00%, specificity = 83.33%; Youden Index = 0.583). The probability (positive predictive value) that a player with values of 55° or below would suffer SP was 83.33%, and the probability (negative predictive value) that a player with values above 55° would experience SP was 75.00% (Figure 5). The 77.78% (14/38) of players with a shoulder internal rotation ROM of 55° or less will suffer from SP in the future (BF_10_ = 27.672 [strong]; log odds ratio = 1.968 [0.570 to 3.366]). Pearson’s chi-square test showed that players with shoulder internal rotation values ROM of 55° or less had an 8.2% higher risk of SP than those badminton players with values above 55° (χ^2^(1) = 8.674, *p* = 0.003, magnitude of association or Cramer’s V effect size 0.478 [moderate]).

## 4. Discussion

To the best of the authors’ knowledge, the present study is the first to predict risk factors for SP in young badminton players competing in the Spanish Championships. Badminton players with SP show lower values of shoulder internal rotation ROM of the dominant side (about 6°) than the contralateral side. In addition, the group of badminton players with SP show lower values ROM of internal rotation in the dominant shoulder than the group of asymptomatic players (about 12°). The mechanics of overhead shuttlecock hitting techniques such as clear, smash and drop cause certain joint adaptations that contribute to injuries and SP [3,17]. These types of strokes, which are repetitive and explosive, primarily stress the adductors and internal rotators of the shoulder during the acceleration phase, followed by increased eccentric activity of the shoulder external rotators during the ‘follow-through’ deceleration phase after the shuttlecock is hit [22]. The eccentric loads or forces generated during these technical overheads striking or throwing actions result in excessive mechanical stress (load divided by tissue cross-section) on the muscle-tendon unit of the rotator cuff, capsule and shoulder ligaments [44]. The repetition of this movement pattern and these high loads contribute to bony adaptations such as torsion of the humerus [45] and soft tissue, resulting in increased ROM of shoulder external rotation and decreased ROM of shoulder internal and total rotation due to shortening or soft tissue injury [46,47,48]. Soft tissue adaptations such as reduced ROM internal and total shoulder rotation have been demonstrated following the acute effects of two consecutive badminton matches [49]. These facts have also been demonstrated in research studies investigating ROM internal and total shoulder rotation in badminton [4,11,50], tennis [46], and cricket [51]. This important risk factor, the deficit in internal and total rotation ROM of the dominant shoulder, termed the ‘glenohumeral internal rotation deficit (GIRD)’, has been associated with injury and SP [46,47,48]. A limitation of shoulder adduction ROM is another common factor in the aetiology of SP [52]. Repetitive explosive stroke from the cocking to the follow-through phases has been shown to cause stress on the epiphyseal plate and overuse epiphyseal injury [53]. In contrast to the other movements, a decrease in shoulder adduction ROM (approximately 7°) was also observed in the group of badminton players with SP in this study, which could contribute to SP in the future. Previous research studies have shown that ROM, especially internal and total shoulder rotation, may decrease as the young player matures physically and gains experience with regular badminton training. The limited internal rotation and shoulder adduction ROM prevalent in throwing athletes leads to altered movement patterns and compensatory movements that increase the prevalence of SP [8,54].

In the same situation, the decreased MIS in non-dominant shoulder extension and MIS imbalance in the shoulder rotators observed in the badminton players in this study. During the badminton stroke movement, the shoulder plays a crucial role as a transmitter of the forces generated by the lower limbs and trunk to the arm and wrist. The repetition of this movement pattern at high velocities leads to changes in the muscle pattern of the shoulder and a heavy load on the shoulder joint tissues [3,55]. Consequently, previous studies have observed that in badminton players there is an imbalance of strength between the internal and external rotators of the shoulder and between both sides of the body [21,22,50]. Specifically, badminton players with SP show greater concentric internal rotation strength than concentric external rotation strength, greater eccentric external rotation strength than concentric external rotation strength, greater concentric internal rotation strength than eccentric external rotation strength [22]. After determining the shoulder strength profile in recreational badminton players, it was found that players on the dominant side had a lower ratio of eccentric external rotation strength to concentric internal rotation strength than those on the non-dominant side [21]. Strength decreases with age, including that of the internal and external rotators of the shoulder. It is possible that testosterone levels, which peak in females at age 17 and in male at age 19 and decline thereafter, explain these results. In this context, a recent study found that adolescent players had higher values for external rotation strength than elite players [44]. The authors therefore conclude that increasing age appears to be associated with greater weakness of the shoulder rotators in elite badminton players. In contrast, other authors did not find these differences in age and competition level in badminton players [50]. However, they observed that the increased strength of the internal rotators on the dominant side in female badminton players was not compensated by the strength of the external rotators, which could make the external rotators vulnerable to large eccentric strength during powerful internal rotation movements on high strokes in badminton. These changes in the strength pattern negatively affect the transmission of strength from the lower limb and trunk to the upper limb [4,11]. When the rotator cuff muscles are unable to centre the humeral head in the glenoid fossa due to muscular imbalance, fatigue or a deficit in motor control can lead to superior translation of the humerus, resulting in impingement of the rotator cuff through the acromion and coracoacromial ligament and subacromial syndrome [15,56,57,58]. In this context, Arora et al. suggest that subacromial syndrome, rotator cuff tendinopathy and scapulothoracic instability or dyskinesia are likely causes of SP in recreational and elite badminton players [3]. Therefore, it is important to analyse an optimal ratio between eccentric antagonist and concentric agonist strength of the dominant shoulder in young badminton players to reduce the risk of injury and SP [3,21].

On the other hand, sagittal spinal curves were not identified as a risk factor for SP in the badminton players in this research study. Previous studies have shown that increased thoracic kyphosis reduces subacromial space and causes subacromial syndrome and SP in the general population [59,60]. However, it is postulated that a thoracic kyphosis greater than 50° is required to reduce subacromial space [59]. The results of the present study are consistent with the scientific literature, although they should be interpreted with caution due to the different population. The values of thoracic kyphosis of badminton players in standing position show values of 44.4°. In the cited studies, no relationship was established between thoracic kyphosis in the seated position and trunk flexion, which is obviously higher. The sagittal spinal alignment of badminton players should be controlled and trained, because a correlation between thoracic hyperkyphosis and shoulder flexion and scapular dyskinesia has been found, risk factors for injuries and SP [60].

According to the second aim of this research study, this is the first study to determine the optimal cut-off value that best distinguishes those badminton players at increased risk for SP. The criterion value of 55° for shoulder internal rotation ROM in young badminton players can help sports professionals to reduce the risk for SP. For sports professionals, this value should be treated as a training target to control the risk for SP in young badminton players. The scientific literature shows normative values for shoulder internal rotation for the dominant side of 42° in competitive youth badminton players [50] and 64° in elite junior players at international level [49]. Higher normative values (70°) have been found in the general population [37]. These normative values should be taken with caution as different methods are used for measurement ROM (position, measuring instrument, control of compensatory movements, lumbar support, end-of-motion criteria, etc.) and as flexibility is specific of age and competitive level [61,62].

Despite potential strengths, the present study shows certain limitations. Based on the fact that SP is multifactorial, several modifiable risk factors have been proposed in addition to limited rotation ROM, such as asymmetric ROM, rotational strength imbalance, weakness of the external rotators, altered scapular kinematics or load changes depending on the technical gesture [63]. The ratio between eccentric antagonist and concentric agonist strength is important when analysing shoulder symptoms experienced by badminton players [53]. Future studies should build predictive models for SP that consider all these modifiable risk factors in performance profiles of badminton players of different ages, competitive levels, and stroke technique [53] as well as a representative sample size. Consensus should also be established on the objective definition of SP and medical diagnosis.

## 5. Conclusions

The young badminton players who had a shoulder internal rotation ROM of 55° or less have a higher risk of SP one year later.

## Figures and Tables

**Table 1:** Differences in shoulder range of motion and maximum isometric strength according to laterality in young badminton players.

Shoulder Variables	Dominant Shoulder	Non-Dominant Shoulder	Bayesian Factor	δ (95% Credible Interval)	Evidence	Total (n = 38)
**Shoulder range of motion (degrees)**						
Flexion *	168.47 ± 18.01	171.42 ± 19.45	6.918	−0.446 (−0.77, −0.12)	Moderate BF_10_	169.95 ± 18.49
Extension	81.58 ± 8.72	85.42 ± 10.11	57.30	−0.581 (−0.92, −0.24)	Very strong BF_10_	83.5 ± 8.75
Abduction	154.37 ± 8.53	157.16 ± 6.40	1.974	−0.354 (−0.678, −0.036)	Anecdotical BF_10_	155.76 ± 6.60
Adduction	149.95 ± 9.31	153.00 ± 8.17	3.427	−0.396 (−0.724, −0.075)	Moderate BF_10_	151.47 ± 7.99
Horizontal abduction	39.21 ± 8.08	40.11 ± 7.84	0.305	−0.164 (−0.474, 0.142)	Moderate BF_01_	39.66 ± 7.55
Internal rotation *	57.58 ± 14.39	63.47 ± 11.36	3.013	−0.386 (−0.713, −0.066)	Moderate BF_10_	60.53 ± 10.86
External rotation *	138.00 ± 15.49	125.97 ± 14.31	3215.1	0.811 (0.442, 1.187)	Extreme BF_10_	128.49 ± 22.39
**Shoulder maximum isometric strength (Newton)**						
Flexion *	204.81 ± 43.90	190.12 ± 51.64	1.205	0.179 (−0.128, 0.490)	Anecdotical BF_10_	197.47 ± 46.02
Extension	267.32 ± 66.97	222.99 ± 53.18	231,963.1	1.046 (0.645, 1.456)	Extreme BF_10_	245.16 ± 56.98
Internal rotation	129.66 ± 31.05	116.80 ± 28.13	86.463	0.605 (0.262, 0.955)	Very strong BF_10_	123.23 ± 27.90
External rotation *	133.09 ± 33.45	132.24 ± 34.44	0.181	0.040 (−0.264, 0.345)	Moderate BF_01_	132.66 ± 32.54

* Variable that changed to logarithmic base (non-parametric data).

**Table 2:** Risk factors between the shoulder pain group and the asymptomatic group.

Variables	Asymptomatic Group (n = 20)	Shoulder Pain Group (n = 18)	Bayesian Factor	δ (95% Credible Interval)	Evidence	Total (n = 38)
**Age, anthropometry and sports background**						
Age (years) *	16.25 ± 0.44	16.27 ± 0.46	0.320	−0.046 (−0.614, 0.512)	Moderate BF_01_	16.26 ± 0.45
Body mass (kg) *	66.95 ± 11.46	65.89 ± 5.63	0.332	0.087 (−0.469, 0.660)	Moderate BF_01_	66.45 ± 9.08
Body height (cm) *	1.77 ± 0.08	1.73 ± 1.14	0.572	0.311 (−0.250, 0.920)	Anecdotical BF_01_	1.76 ± 0.11
Body mass index (kg/m^2^) *	21.14 ± 2.79	22.40 ± 4.92	0.461	−0.245 (−0.843, 0.311)	Anecdotical BF_01_	21.74 ± 3.94
Badminton experience (years) *	6.15 ± 1.73	6.61 ± 1.94	0.399	−0.192 (−0.781, 0.363)	Anecdotical H_01_	6.37 ± 1.82
Weekly training volume (h) *	9.78 ± 6.42	9.75 ± 4.67	0.315	0.003 (0.559, 0.566)	Moderate BF_01_	9.76 ± 5.59
Weekly training frequency (days) *	3.90 ± 1.21	4.00 ± 0.91	0.326	−0.070 (−0.641, 0.487)	Moderate BF_01_	3.95 ± 1.06
Training sessions duration (h) *	2.27 ± 0.82	2.22 ± 0.54	0.322	0.057 (−0.501, 0.625)	Moderate BF_01_	2.25 ± 0.70
**Sagittal spinal curves (degree)**						
SSP thoracic curve	46.20 ± 11.01	46.89 ± 7.43	0.322	−0.055 (−0.623, 0.503)	Moderate BF_01_	46.53 ± 9.37
SSP lumbar curve *	9.30 ± 6.97	14.00 ± 11.44	0.799	−0.395 (−1.019, 0.175)	Anecdotical BF_01_	11.53 ± 9.52
RSP thoracic curve	40.9 ± 11.38	44.44 ± 9.86	0.475	−0.255 (−0.854, 0.302)	Anecdotical BF_01_	42.58 ± 10.69
RSP lumbar curve	31.00 ± 7.72	30.56 ± 8.62	0.319	0.041 (−0.518, 0.608)	Moderate BF_01_	30.79 ± 8.05
TFP thoracic curve	63.60 ± 10.17	62.11 ± 9.88	0.342	0.113 (−0.443, 0.689)	Anecdotical BF_01_	62.90 ± 9.93
TFP lumbar curve *	26.80 ± 8.95	32.56 ± 12.64	0.885	−0.419 (−0.1046, 0.155)	Anecdotical BF_01_	29.52 ± 11.10
**Shoulder range of motion (degree)**						
Flexion DS *	172.60 ± 11.86	163.89 ± 22.50	0.770	0.386 (−0.183, 1.008)	Anecdotical BF_01_	168.47 ± 18.01
Flexion N-DS *	174.70 ± 12.13	167.78 ± 25.15	0.506	0.275 (−0.283, 0.878)	Anecdotical BF_01_	171.42 ± 19.45
Extension DS	82.90 ± 10.71	80.11 ± 4.47	0.477	0.257 (−0.301, 0.856)	Anecdotical BF_01_	81.58 ± 8.37
Extension N-DS	87.10 ± 11.92	83.56 ± 7.53	0.499	0.271 (−0.287, 0.873)	Anecdotical BF_01_	85.42 ± 10.11
Abduction	157.25 ± 7.20	154.11 ± 5.59	0.746	0.379 (−0.189, 1.000)	Anecdotical BF_01_	155.76 ± 6.60
Adduction DS	153.20 ± 9.14	146.33 ± 8.29	2.859	0.645 (0.034, 1.308)	Anecdotical BF_01_	149.94 ± 9.31
Adduction N-DS	154.60 ± 7.49	151.22 ± 8.74	0.600	0.324 (−0.238, 0.935)	Anecdotical BF_01_	153.00 ± 8.17
Horizontal abduction	39.90 ± 6.85	39.38 ± 8.45	0.321	0.051 (−0.508, 0.618)	Moderate BF_01_	39.66 ± 7.55
Internal rotation DS *	63.20 ± 17.00	51.33 ± 7.03	5.334	0.748 (0.117, 1.426)	Moderate BF_10_	57.58 ± 14.39
Internal rotation N-DS *	66.20 ± 1.89	60.44 ± 10.21	0.843	0.407 (−1.164, 1.033)	Anecdotical BF_01_	63.47 ± 11.35
External rotation DS *	138.10 ± 14.59	137.89 ± 16.86	0.316	0.010 (−0.551, 0.574)	Moderate BF_01_	138.00 ± 15.49
External rotation N-DS *	124.30 ± 17.69	127.83 ± 9.42	0.395	−0.187 (−0.775, 0.367)	Moderate BF_01_	125.97 ± 14.31
**Shoulder range of motion asymmetry (degree)**						
Flexion	4.70 ± 4.37	5.00 ± 5.33	0.320	−0.047 (−0.614, 0.512)	Moderate BF_01_	4.84 ± 4.78
Extension	6.20 ± 5.02	4.78 ± 4.40	0.441	0.230 (−0.326, 0.825)	Moderate BF_01_	5.53 ± 4.73
Abduction	6.90 ± 5.49	5.33 ± 3.76	0.473	0.254 (−0.303, 0.853)	Moderate BF_01_	6.16 ± 4.75
Adduction	6.20 ± 5.06	6.00 ± 4.55	0.317	0.031 (−0.528, 0.597)	Moderate BF_01_	6.11 ± 4.76
Horizontal abduction	5.00 ± 3.28	3.00 ± 2.40	1.771	0.559 (−0.036, 1.209)	Anecdotical BF_10_	4.05 ± 3.03
Internal rotation	12.00 ± 11.59	10.22 ± 9.23	0.351	0.128 (−0.427, 0.706)	Anecdotical BF_01_	11.16 ± 10.43
External rotation	20.40 ± 28.30	13.38 ± 9.68	0.467	0.249 (−0.308, 0.847)	Anecdotical BF_01_	17.08 ± 21.61
**Shoulder maximum isometric strength (Newton)**						
Flexion DS *	206.91 ± 55.32	202.49 ± 27.62	0.327	0.075 (−0.482, 0.646)	Moderate BF_01_	204.82 ± 43.90
Flexion N-DS *	198.35 ± 67.06	180.98 ± 24.70	0.481	0.259 (−0.289, 0.859)	Anecdotical BF_01_	190.12 ± 51.64
Extension DS	277.01 ± 15.77	256.56 ± 62.98	0.446	0.234 (−0.322, 0.829)	Anecdotical BF_01_	267.32 ± 66.96
Extension N-DS	239.81 ± 55.65	204.32 ± 44.65	1.848	0.567 (−0.030, 1.218)	Anecdotical BF_10_	223.00 ± 53.18
Internal rotation DS	130.09 ± 3085	129.19 ± 32.16	0.316	0.021 (−0.539, 0.586)	Moderate BF_01_	116.80 ± 28.14
Internal rotation N-DS	118.50 ± 30.88	114.92 ± 25.49	0.335	0.095 (−0.461, 0.669)	Anecdotical BF_01_	123.23 ± 27.89
External rotation *	134.52 ± 38.78	130.60 ± 24.83	0.333	0.090 (−0.466, 0.663)	Anecdotical BF_01_	132.66 ± 32.54
**Maximum isometric strength imbalance (Newton)**						
Flexion	24.28 ± 17.58	25.34 ± 17.98	0.320	−0.045 (−0.612, 0.514)	Moderate BF_01_	24.78 ± 17.53
Extension	44.65 ± 40.37	52.24 ± 30.11	0.373	−0.161 (−0.744, 0.394)	Anecdotical BF_01_	48.24 ± 35.61
Internal rotation	23.78 ± 10.14	17.12 ± 12.13	1.170	0.478 (−0.104, 1.116)	Anecdotical BF_01_	20.62 ± 11.48
External rotation	13.85 ± 15.17	14.68 ± 10.20	0.320	−0.049 (−0.616, 0.510)	Anecdotical BF_01_	14.24 ± 12.89

* Variable that changed to logarithmic base (non-parametric data); SSP: slumped sitting posture, RSP: relaxed standing posture; TFP: maximum trunk forward flexion posture; DS: dominant shoulder; N-DS: non-dominant shoulder.

**Table 3:** Predictor variables of shoulder pain in young badminton players.

Shoulder Variables	Estimation	Standard Error	Odds Ratio	z	*p* Value	95% Confidence Interval
ROM adduction DS	−0.067	0.060	1.069	−1.111	0.267	−0.185, 0.051
ROM internal rotation DS	−0.116	0.055	1.122	−2.110	0.035	−0.224, −0.008
ROM horizontal abduction	−0.044	0.064	1.045	−0.693	0.488	−0.169, 0.081
MIS extension N-DS	−0.010	0.010	1.010	−1.049	0.294	−0.029, 0.009
MIS internal rotation imbalance	−0.042	0.037	1.042	−1.132	0.258	−0.115, 0.031

ROM: range of motion; MIS: maximum isometric strength; DS: dominant shoulder; N-DS: non-dominant shoulder.

## Data Availability

The data associated with the paper are not publicly available but are available from the corresponding author on reasonable request.
